# Effects of Post-Curing on Mechanical Strength and Cytotoxicity of Stereolithographic Methacrylate Resins

**DOI:** 10.3390/polym17152132

**Published:** 2025-08-02

**Authors:** Alfredo Rondinella, Matteo Zanocco, Alex Lanzutti, Wenliang Zhu, Enrico Greco, Elia Marin

**Affiliations:** 1 Department Polytechnic of Engineering and Architecture, University of Udine, 33100 Udine, Italy; alfredo.rondinella@uniud.it (A.R.);; 2 Ceramic Physics Laboratory, Faculty of Materials Science and Engineering, Kyoto Institute of Technology, Sakyo-ku, Matsugasaki, Kyoto 606-8585, Japan; wlzhu@kit.ac.jp; 3 Institute for the Advanced Study of Culture and the Environment (IASCE), University of South Florida, 4202 E. Fowler Ave, Tampa, FL 33620, USA; 4 National Interuniversity Consortium of Materials Science and Technology (INSTM), Via G. Giusti 9, 50121 Firenze, Italy; 5 Biomaterials Engineering Laboratory, Faculty of Materials Science and Engineering, Kyoto Institute of Technology, Sakyo-ku, Matsugasaki, Kyoto 606-8585, Japan; 6 Biomedical Research Center, Kyoto Institute of Technology, Sakyo-ku, Matsugasaki, Kyoto 606-8585, Japan

**Keywords:** stereolithographic resin, PMMA, curing, mechanical properties, cytotoxicity, Raman spectroscopy

## Abstract

This study investigated the influence of curing temperature and time on both the mechanical properties and cytotoxicity of stereolithographic polymethyl methacrylate (PMMA) resin. After printing using stereolithographic equipment, the resin was cured at 45 °C, 60 °C, and 75 °C for up to 120 min. Our results reveal that the mechanical properties achieved a peak after approximately 30 min of curing at the two highest temperatures, followed by a subsequent decrease, while curing at 45 °C resulted in a constant increase in mechanical properties up to 120 min. Testing with *S. epidermidis* and *E. coli* exhibited a bland antibacterial effect, with the number of living bacteria increasing with both the time and temperature of curing. To assess potential cytotoxicity, the materials were also tested with human *fibroblasts*, and the trends observed were similar to what was previously seen for both bacteria strains. Interestingly, an association was observed between the intensity ratio of two Raman bands (around 2920 and 2945 cm^−1^), indicative of long-PMMA-chain formation and cytotoxicity. This finding suggests that Raman spectroscopy has the potential to serve as a viable method for estimating the cytotoxicity of 3D printed PMMA objects.

## 1. Introduction

Three-dimensional (3D) printing technology has undergone phenomenal growth in recent years, finding applications in diverse fields ranging from rapid prototyping [1] and personalized medicine [2] to aerospace [3] and construction [4]. Among the various additive manufacturing techniques, resin-based 3D printing techniques, stereolithography, and digital light processing in particular [5] offer several advantages, including the possibility to build extremely complex shapes, high resolution, the ability to achieve smooth surface finish, and the possibility to use a wide range of available materials [6]. This technology is also readily accessible, with printers suitable for both industrial and consumer settings.

Methyl methacrylates, commonly known as acrylics, are the most prevalent resin employed in vat polymerization processes due to their transparency, excellent mechanical properties, and compatibility with various post-processing techniques [7]. However, VAT resins exist in numerous formulations, each tailored for specific functionalities. For instance, certain resins are biocompatible, ideal for medical applications and, in particular, for denture bases and aligners used in the dental field, while others are formulated to provide enhanced flexibility or increased strength [8].

Despite their significant advantages, resin-based 3D printing technologies also raise concerns regarding potential health risks. Compared to filament-based techniques like Fused Filament Fabrication (FFF) [9,10] and other melting techniques, where the primary hazard is particle inhalation from heated filaments, resin-based processes involve uncured or partially cured liquid resins, which can be irritating to the skin and eyes upon contact and potentially pose respiratory risks if inhaled [11,12]. This is particularly concerning because polymethyl methacrylate (PMMA) is produced through the polymerization of methyl methacrylate (MMA), a known irritant and suspected respiratory sensitizer [13].

In stereolithography and digital light processing, the most common resin-based 3D printing techniques, a laser beam selectively cures layers of liquid resin, transforming MMA monomers into PMMA polymer chains [14]. However, if curing parameters are not properly optimized, incomplete polymerization may occur, leaving residual MMA within the printed object. Uncured MMA can leach out over time, posing a potential health hazard [15,16].

UDMA/PMMA resins, in particular, are a class of materials formed by combining urethane dimethacrylate (UDMA) and PMMA [17,18]. UDMA offers superior strength, durability, and rapid light curing [17,19], while PMMA contributes biocompatibility, ease of processing, and esthetics. Blending these materials addresses the limitations of each individual component. The resulting UDMA/PMMA resin exhibits enhanced mechanical properties, including improved strength, toughness, and wear resistance, while retaining favourable handling characteristics and esthetics. These features allow for the tailoring of material properties to suit specific biomedical applications in the dental field, such as denture base materials, dental restorations, and luting cements [20].

Unfortunately, accurately determining the degree of polymerization in a non-destructive manner remains a significant challenge. Conventional methods often involve destructive testing or specialized equipment [21,22,23]. In this context, Raman spectroscopy emerges as a promising tool. As a non-destructive analytical technique, Raman spectroscopy enables the evaluation of material composition and the detection of residual MMA by detecting specific chemical bonds [24,25,26]. By understanding the correlation between the Raman spectra and the degree of polymerization, it becomes possible to assess the potential health risks associated with 3D printed PMMA components.

Given the growing concerns regarding the health implications of resin-based 3D printing, this study aims to investigate the influence of curing parameters on the mechanical properties, cytotoxicity, and degree of polymerization of PMMA resins. The main objective of this work is to establish a correlation between curing conditions and the material performance and biocompatibility. We employed Raman spectroscopy to assess the potential health risks associated with 3D printed PMMA objects based on their chemical structure.

## 2. Materials and Methods

### 2.1. Materials

Samples were produced using a commercial stereolithographic 3D printer with a 405 nm laser source (Foam2, Formlabs, Somerville, MA, USA) with a nominal resolution of 25 μm. The resin (Flexible Resin, Formlabs, Somerville, MA, USA) was composed of UDMA (≥50–≤70%), MMA (≥30–≤40%), and diphenyl (2,4,6-trimethylbenzoyl) phosphine oxide (≤0.9%) as a photoinitiator, and its detailed composition is a trademark secret of the producer.

### 2.2. Sample Preparations

The stereolithographic process was performed at 31 °C, with a nominal layer resolution of 50 μm. After printing, samples were washed in isopropyl alcohol (KT Chemicals Co., Ltd., Osaka, Japan) for 20 min (Form Wash, Formlabs, Somerville, MA, USA). Samples were UV-cured at different temperatures (45, 60, and 75 °C) for up to 120 min using dedicated equipment (Form Cure, Formlabs, Somerville, MA, USA). It should be noted that the recommended producer’s indications for UV curing of this resin are 10 min at 60 °C to maximize tensile strength.

### 2.3. Characterization Techniques

#### 2.3.1. Confocal Imaging

Prior to observation, the samples were cleaned in an ultrasonic bath containing ethanol for 5 min to remove surface contaminants. All samples were then mounted with the same orientation to minimize the influence of printing direction and other processing-related variables. Micrographs of the sample surfaces were taken using a 3D laser-scanning confocal microscope (VKX200K series, Keyence, Osaka, Japan) with magnifications including 10×, 50×, and 150× and a numerical aperture between 0.30 and 0.95. Surface maps obtained from merging of different images could be acquired using a dedicated automated xy stage combined with the autofocus function for the z axis. For each sample, five images were captured at each magnification.

#### 2.3.2. Raman Spectroscopy

Raman spectroscopy was conducted utilizing a confocal laser Raman microscope (RAMANtouch, Nanophoton Co., Ltd., Osaka, Japan) with excitation sources at 532 nm and a nominal power of 200 mW. To mitigate the risk of sample burning, the power output was regulated by adjusting a dedicated Neutral Density (ND) filter. The micro-probe employed lenses ranging from 5× to 100× magnification, with numerical apertures spanning from 0.5 to 0.23. The average spectra for each material were then analyzed and deconvoluted using a dedicated software (Labspec 5.0, Horiba, Kyoto, Japan).

#### 2.3.3. FTIR

Fourier Transform Infrared Spectroscopy (FTIR) was employed to characterize the samples using an FTIR-4000 spectrometer (JASCO, Tokyo, Japan), equipped with a Michelson interferometer (28° configuration) and corner-cube mirrors. Spectra were acquired through a 200 × 200 um^2^ aperture with an acquisition time of 30 s per scan. The analysis covered the spectral range of 200–4000 cm^−1^. Data collection was performed using Spectra Manager software (JASCO), while signal processing and spectral deconvolution were carried out with Origin 8.5 (OriginLab Corp., Northampton, MA, USA) and LabSpec 5 (Horiba, Kyoto, Japan).

#### 2.3.4. X-Ray Diffraction

X-ray Diffraction (XRD) analyses were performed on a Rigaku Ultima IV (Rigaku Corporation, Tokyo, Japan), using the CuKa radiation. Diffraction patterns were acquired in the range of 5–80° with a step size of 0.02 at a rate of 3°/min. The penetration depth of the XRD probe was in the order of 1 mm. For PMMA, the peaks at about 19° [002] and 30° [211] were the only ones clearly visible [27]. A secondary, crystalline phase, located at 19.7°, 24.3°, and 37.4°, could not be identified. The intensity of the diffraction peaks related to the secondary phase was influenced by the exposure to UV light and temperature during post-curing.

#### 2.3.5. Mechanical Testing

Mechanical properties of the different scaffolds were measured using a tensile tester (STA-1150, ORIENTEC, Tokyo, Japan). The samples, specifically prepared for mechanical testing, featured two reinforced clamping regions on both sides and had an initial testing length of 20 mm. The specimens were then stretched at a tensile rate of 10 mm/min at room temperature. The tensile strength, elongation at break, and tensile modulus were given by the software used by the tensile tester after obtaining the stress–strain curve, while the toughness was calculated using commercial software (Origin 8.5, Originlab Corp., Northampton, MA, USA). The measurements were repeated six times for each group of samples.

### 2.4. Biological Testing

#### 2.4.1. Cell Testing

*Normal human dermal fibroblast* (HDF, CA10605a) cells derived from a 22-year-old female were purchased from Toyobo Life Science (Osaka, Japan). HDF cells were cultured in Dulbecco’s modified Eagles’ medium (DMEM) containing phenol red, supplemented with 10% *v*/*v* fetal bovine serum (FBS), 1% MEM nonessential amino acid solution, 1% L-sodium pyruvate, and 1% penicillin—streptomycin mixed solution (complete medium) in a humidified incubator at 37 °C and under 5% CO_2_ conditions.

Cell viability quantification was performed using an MTT assay (MTT cell count kit, Nacalai Tesque, Kyoto, Japan), which is based on the cleavage of a tetrazolium salt using metabolically active cells to form a water-insoluble formazan dye. To adapt this method for testing 3D printed PMMA discs, HDF cells were initially seeded in 24-well plates at a density of 2 × 10^4^ cells per well and incubated overnight to allow cell attachment. After incubation, sterilized 3D printed PMMA discs were carefully placed in each well, ensuring good contact with the cell monolayer. The cells were then incubated with the PMMA discs for designated periods (48 h) to assess both short-term and long-term cell viability. At the end of the treatment period, the PMMA discs were removed, and the medium from each well was aspirated. The cells were washed once with PBS to remove any residual PMMA material or secreted factors. Subsequently, 500 μL of complete culture media and 50 µL of the MTT solution were added to each well, and the cells were incubated for 3 h at 37 °C. After incubation, 500 μL of the solubilization solution was added to each well to dissolve the precipitated formazan via gentle pipetting. Two 100 μL aliquots from each well were then collected and transferred to a 96-well plate, and the absorbance at 550 nm was measured using an Infinite F50 Plus microplate reader (Tecan, Männedorf, Switzerland). This approach ensures that the PMMA discs’ impact on cell viability was accurately assessed.

#### 2.4.2. Bacteria Culture

*Escherichia coli* (25922^®^ATCC™) (simply *E. coli*, henceforth) and *Staphylococcus epidermidis* (*S. epidermidis*) were cultured at 37 °C using brain heart infusion (BHI) agar (Nissui, Tokyo, Japan). Starting from an initial 1.0 × 10^9^ CFU/mL, the concentration was diluted with phosphate-buffered saline (PBS) at physiological pH and ionic strength. Subsequently, 100 μL of the bacterial suspension at a density of 1 × 10^8^ CFU/mL was spread onto a BHI agar plate. The samples were sterilized by UV and pressed into the bacteria on BHI agar for inoculation.

After 24 h of incubation, the samples were washed with PBS, and bacterial viability was assessed using a colorimetric assay (Microbial Viability Assay Kit-WST, Dojindo, Kumamoto, Japan). This assay employed a colorimetric indicator (WST-8), which produces a water-soluble formazan dye upon reduction in the presence of an electron mediator. The amount of the formazan dye generated is directly proportional to the number of living microorganism. Solutions were analyzed using microplate readers (EMax, Molecular Devices, Sunnyvale, CA, USA) through optical density at 600 nm (OD600).

### 2.5. Statistical Analysis

To assess the statistical significance of the differences observed among the groups, we employed a one-way analysis of variance (ANOVA). This approach enabled us to determine whether any of the mean values for the measured outcomes varied significantly across the experimental groups. For each test, we utilized a sample size of *n* = 5 specimens, ensuring adequate statistical power for the analysis.

## 3. Results

**Figure 1:**
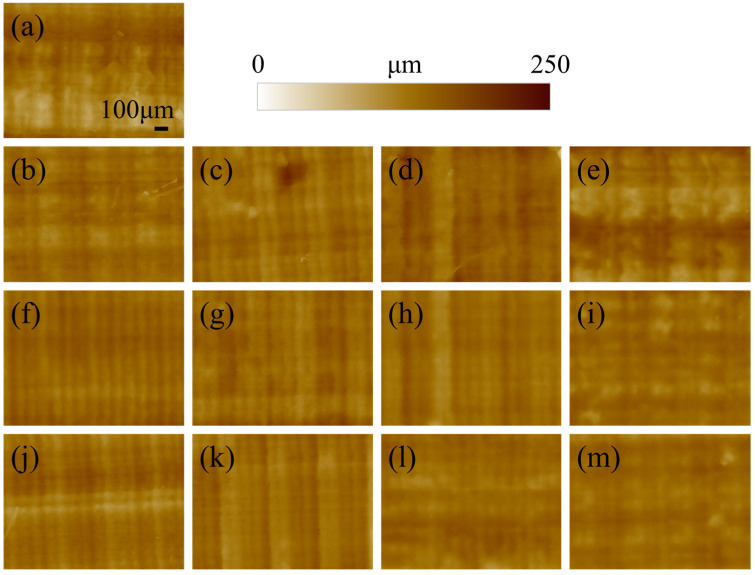
Surface morphology at low magnifications for the different specimens as a function of the time and temperature of post-curing: (**a**) no post-curing; (**b**–**e**) 45 °C for 10, 30, 60, and 120 min; (**f**–**i**) 60 °C for 10, 30, 60, and 120 min; (**j**–**m**) 75 °C for 10, 30, 60, and 120 min.

**Figure 2:**
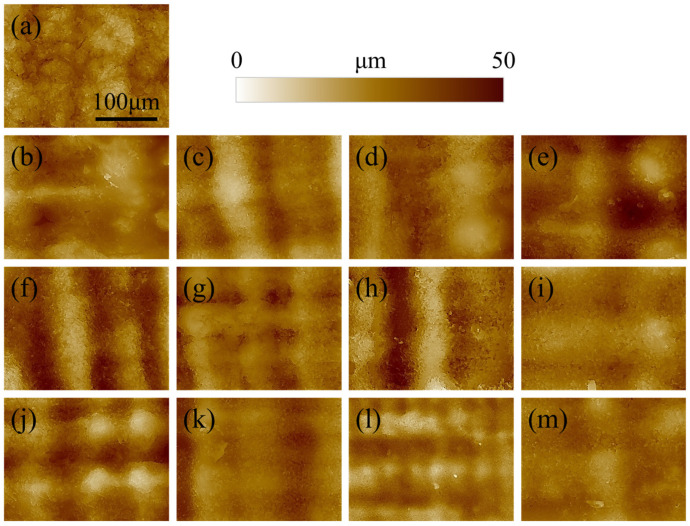
Surface morphology at high magnifications for the different specimens as a function of the time and temperature of post-curing: (**a**) no post-curing; (**b**–**e**) 45 °C for 10, 30, 60, and 120 min; (**f**–**i**) 60 °C for 10, 30, 60, and 120 min; (**j**–**m**) 75 °C for 10, 30, 60, and 120 min.

The morphology of the surface of the printed specimen as a function of post-curing time and temperature is presented in Figure 1. For short post-curing times (10 and 30 min) increasing the printing temperature does not seem to affect the roughness, as the layering marks are clearly visible and regular. When the time is extended to 60 or 120 min, the lines associated with layering became more blurred and undistinguished, meaning that the curing is directly affecting the surface morphology. This results in a smoothing of the surface roughness, which is more evident for images acquired at higher magnifications (Figure 2).

The surface of the reference non-post-cured sample of Figure 2a appears more granular and irregular when compared to the other materials. This is due to the relatively low geometrical stability of the resin, which is not completely polymerized and sticky. As a result, the sample is subject to creep, which affects the surface morphology. For the other samples, the roughness clearly decreases with increasing post-curing time and temperature, resulting in a smoother surface.

**Figure 3:**
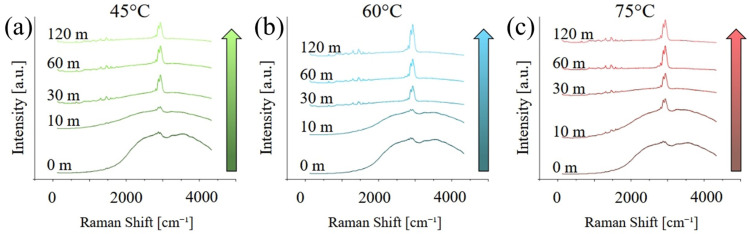
Raman spectra acquired on the different samples, in the range between 100 and 4250 cm^−1^, as a function of the post-curing temperature: (**a**) 40 °C, (**b**) 60 °C, and (**c**) 75 °C.

Figure 3 presents the Raman spectra acquired on the different samples, in the range between 100 and 4250 cm^−1^. At short curing times, the spectra are dominated by a broad, fluorescence band between 1500 and about 4500 cm^−1^, which is caused by the residual presence of the photoinitiator with maybe a minor contribution for un-polymerized monomers, in particular MMA. The fluorescence progressively disappears, and its contribution is almost negligible after 120 min of post-curing. Peaks related to PMMA, in particular between 500 and 1800 cm^−1^ and again between 2700 and 3200 cm^−1^, appear to be more intense for higher post-curing temperatures.

**Figure 4:**
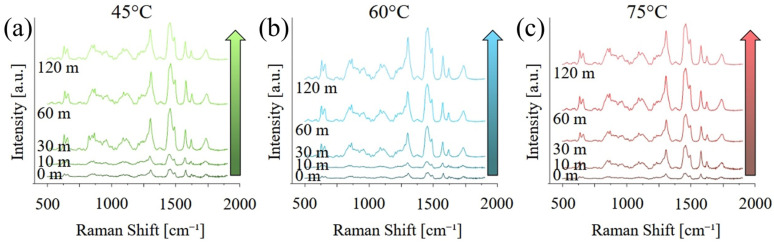
Raman spectra acquired on the different samples, in the range between 500 and 2000 cm^−1^, as a function of the post-curing temperature: (**a**) 40 °C, (**b**) 60 °C, and (**c**) 75 °C.

Figure 4 shows the same graphs as Figure 3 but focused on the region between 500 and 200 cm^−1^ and after baseline removal. As previously observed, the intensity of the bands related to PMMA and UDMA increase with both curing temperature and time, indicating an increase in the degree of both polymerization and cross-linking. A list of the main vibrational modes, their position, and a relative literature reference can be found in Table 1.

**Figure 5:**
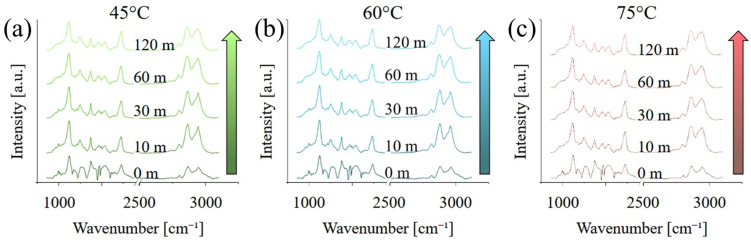
FTIR spectra acquired on the different samples, in the range between 900 and 3200 cm^−1^, as a function of the post-curing temperature: (**a**) 40 °C, (**b**) 60 °C, and (**c**) 75 °C.

FTIR spectra acquired on the samples in the region between 900 and 3200 cm^−1^ are presented in Figure 5. Unlike the Raman spectra of Figure 3 and Figure 4, FTIR spectra are not affected by the fluorescence, and all spectra have the same general appearance, independently from the curing time and temperature. A list of the main vibrational modes, their position, and a relative literature reference can be found in Table 2.

The most relevant differences between the various FTIR spectra are the intensity of the peak at about 1720 cm^−1^, associated with C=O vibrations, and the relative intensity of the peak at about 2945 cm^−1^ with respect to the main peak located at about 2960 cm^−1^. The former is indicative of the enhanced formation and stabilization of carbonyl groups within the polymer network, as a result of increased cross-linking. Similarly, the latter is also caused by the polymerization and cross-linking of the resin, as the number of residual CH_3_ groups progressively decreases in favour of CH_2_.

**Figure 6:**
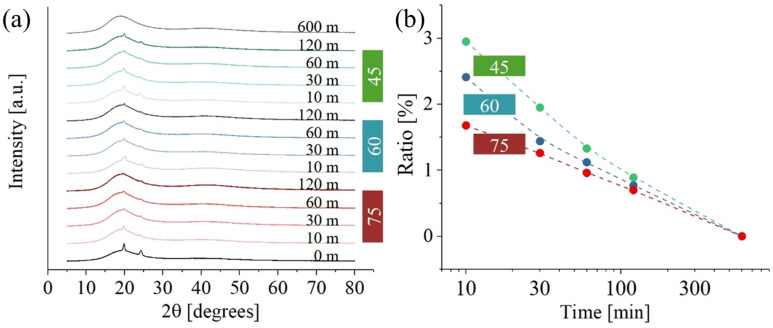
XRD data (**a**) and crystalline ratio (**b**) as a function of post-curing time and temperature (green, 45 °C; blue, 60 °C; and red, 75 °C).

Figure 6 shows XRD data for PMMA. While Figure 6a illustrates results at different temperatures and curing times, Figure 6b gives the ratio between the crystalline and amorphous phase. Crystalline peaks at about 19° [002] and 30° [211] were the only ones clearly visible (31). A secondary crystalline phase, located at 19.7°, 24.3°, and 37.4°, could not be identified. The intensity of the diffraction peaks related to the secondary phase is clearly influenced by the exposure to UV light and temperature during post-curing. As previously stated, the exact composition of the resin is not available, suggesting the possibility that resin components include additives with a certain degree of crystallinity. While the precise nature of these additives remains undetermined, it could be hypothesized that they are low-molecular-weight compounds used for viscosity regulation. The presence of these additives was not detected in either the Raman or FTIR spectra, likely because their signals are masked by the more intense signals of UDMA and PMMA, which are present in higher concentrations. However, their presence is detectable in the XRD spectra due to the crystallinity of these components. Regardless of these additives’ specific composition, Figure 6a shows a consistent decrease in the presence of these peaks with increased post-curing time. This trend suggests that prolonged exposure to UV light may cause the degradation of such additives.

The curve at 600 m was acquired at room temperature and clearly shows that, for very long times, the crystalline phase of the material drops. Figure 6b illustrates the ratio of crystalline phase to amorphous phase as a function of post-curing time at various temperatures. Initially, the ratio is higher at lower temperatures for shorter post-curing times. However, as the post-curing time increases, this ratio converges to a temperature-independent value. One possible explanation for this observation is that, beyond a certain time threshold, the influence of temperature becomes negligible compared to the impact of UV exposure.

**Figure 7:**
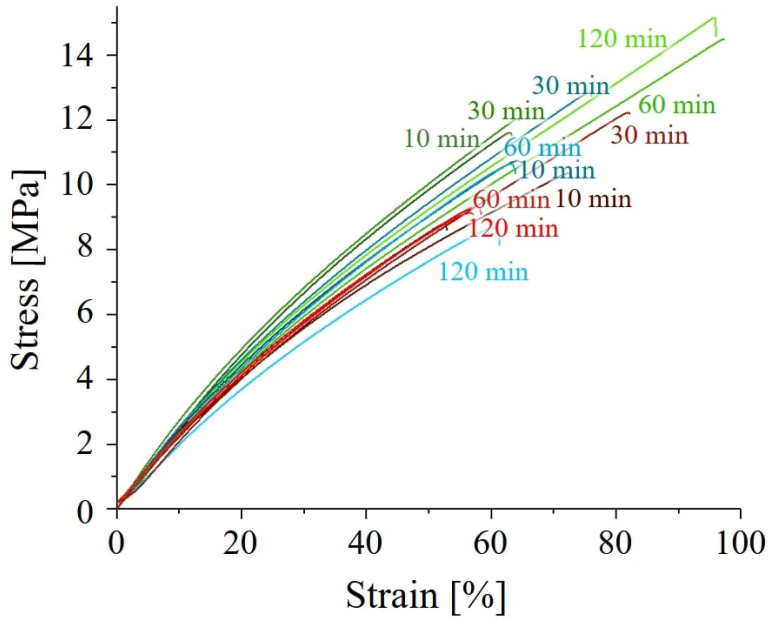
Representative stress–strain curves for the different samples, as a function of post-curing time and temperature (green, 45 °C; blue, 60 °C; and red, 75 °C).

**Figure 8:**
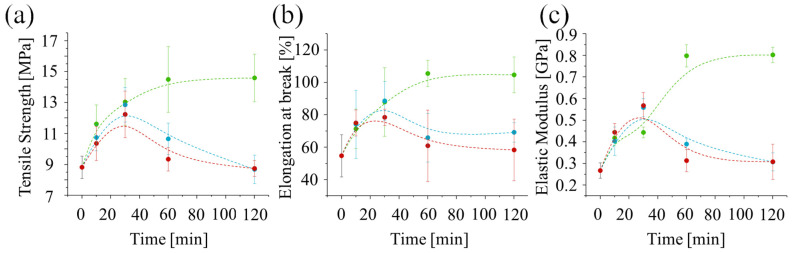
Mechanical properties obtained from the tensile testing of the specimen, (**a**) ultimate strength, (**b**) strain at break, and (**c**) elastic modulus, as a function of post-curing time and temperature (green, 45 °C; blue, 60 °C; and red, 75 °C).

Representative tensile stress–strain curves for all polymers are presented in Figure 7. The general shape of the stress–strain curves for all post-curing conditions is similar, indicating that embrittlement was not induced by the curing process. Post-curing time and temperature significantly impact the mechanical properties of the resin. Samples cured for longer durations (60 and 120 min) at the lowest temperature (45 °C) exhibited the best performance. Conversely, post-curing at higher temperatures (60 °C or 75 °C) for extended times (120 min) resulted in poorer mechanical properties. In fact, all post-curing processes at 75 °C led to a decrease in performance. This suggests that lower temperatures are always preferable for post-curing this resin. A more detailed report of the mechanical performances as a function of post-curing time and temperature is presented in Figure 8.

In all cases, the ultimate strength initially increases with treatment time up to around 30 min. However, for post-curing at higher temperatures (60 °C or 75 °C), the ultimate strength (Figure 8a), strain at break (Figure 8b), and elastic modulus (Figure 8c) start to decrease after 30 min. Interestingly, the values of these properties at 30 min are similar for all three post-curing temperatures, suggesting that UV irradiation alone is responsible for the strengthening. The decrease observed after 30 min for higher temperatures suggests that a thermally induced degradation process may be occurring. This could be due to thermal oxidation, over-crosslinking, or chain scission.

**Figure 9:**
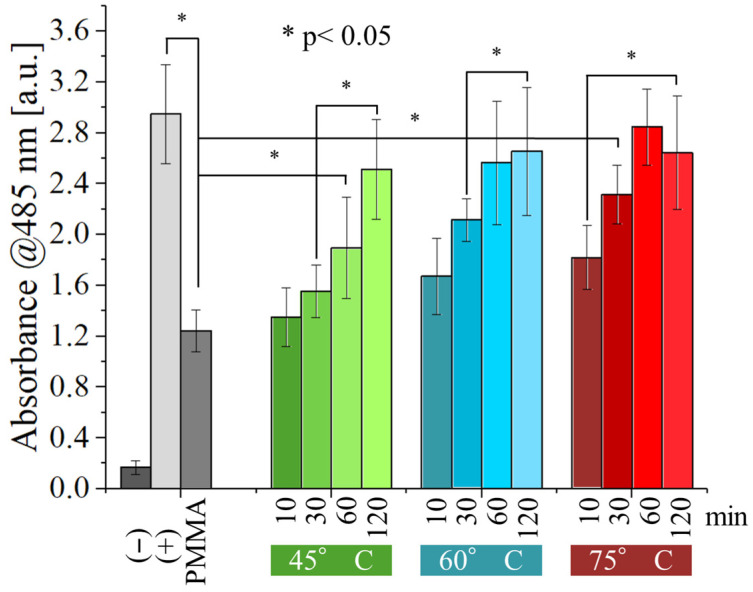
WST-8 results for the different samples tested again with *E. coli*, as a function of post-curing time and temperature.

**Figure 10:**
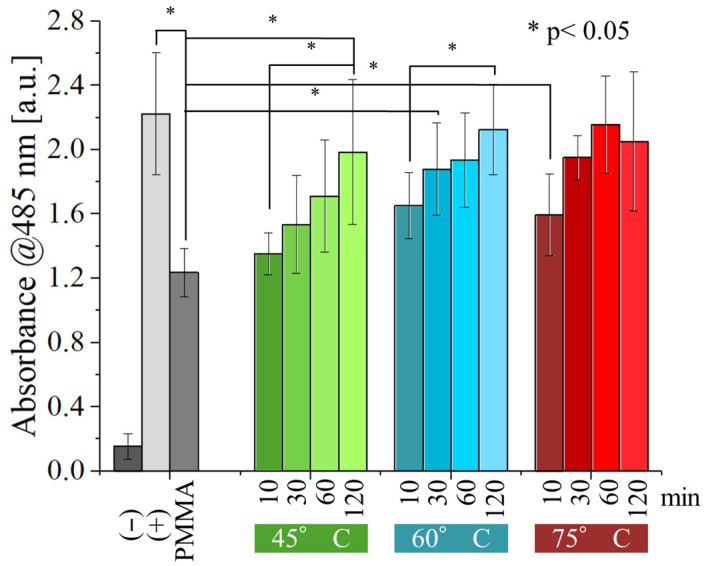
WST-8 results for the different samples tested again with *S. epidermidis*, as a function of post-curing time and temperature.

Figure 9 and Figure 10 show the results of the WST-8 test performed with *E. coli* and *S. epidermidis* on the samples, as a function of both post-curing time and temperature and compared with the non-post-cured resin.

The non-post-cured PMMA sample exhibited a weak antibacterial effect against *E. coli* (Figure 9). However, this effect was statistically significant compared to the negative control, indicating minimal inherent antibacterial properties. Interestingly, post-curing appears to negate this effect. As post-curing time and temperature increase, the number of viable bacteria progressively rises. Samples post-cured for at least 60 min at 60 °C or higher displayed no statistically significant difference from the positive control, indicating a complete loss of antibacterial activity. At the lower temperature of 45 °C, the weak antibacterial effect persisted for 60 min but ultimately vanished after 120 min of post-curing.

Compared to *E. coli*, the antibacterial activity of the resin tested against *S. epidermidis* (Figure 10) is inferior, with post-curing of 30 min or more at 60 and 75 °C showing no statistically significant difference from the positive control. For post-curing performed at 45 °C, the same result can only be achieved after about 120 min of post-curing. These results indicate that *S. epidermidis* has a higher resistance to the antibacterial effect of the resin.

**Figure 11:**
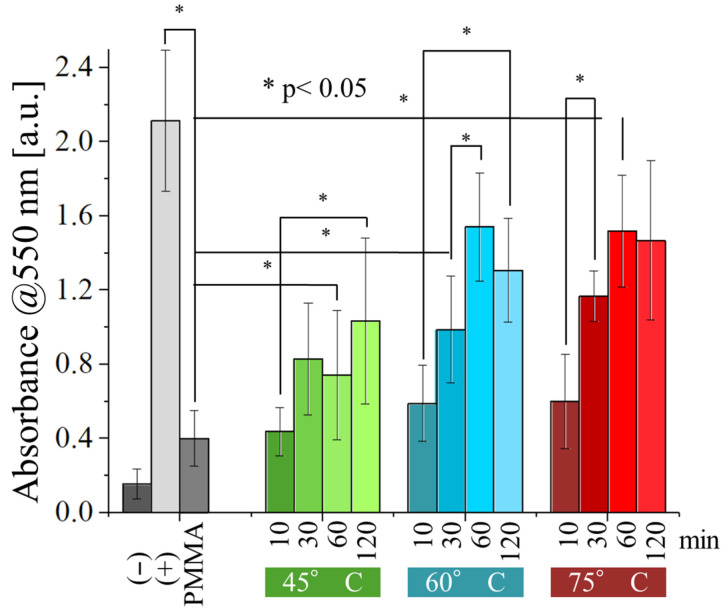
MTT assay results (absorbance at 550 nm) for the different samples, as a function of the temperature and time of post-curing.

The results of the viability test performed with normal human dermal fibroblasts (Figure 11) revealed that the resins are far more cytotoxic to human cells than they are to bacteria. Even after 120 min of post-curing, the absorbance measured on the samples was significantly lower compared to the one observed for the positive control, at all testing conditions. As observed before for bacteria, absorbance does increase with the time and temperature of post-curing, but the effect is less noticeable, suggesting that post-curing alone is not sufficient to make them into suitable biomaterials.

## 4. Discussion

Optimizing the curing parameters of UV-curable resins in 3D printing can improve mechanical resistance and durability. However, these parameters also have a significant effect on the cytocompatibility and on the antibacterial properties of these materials. For instance, we observed that under-cured resins exhibit a beneficial antibacterial effect (Figure 9 and Figure 10). Unfortunately, this also translates to a cytotoxic effect against human cells (dermal fibroblasts in our experiments). Such cytotoxicity poses a significant health risk, especially for applications involving prolonged internal or oral contact, such as dental restorations, teeth retainers, or even tissue engineering. While the risk might be negligible for external applications, it becomes a major concern for these internal or oral uses.

**Figure 12:**
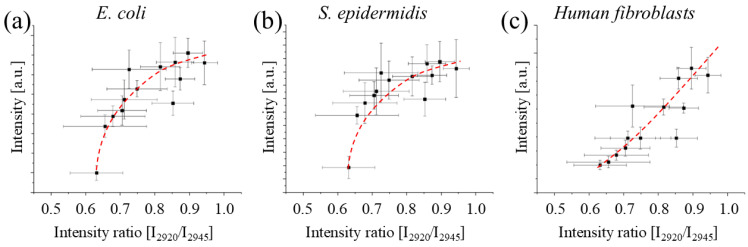
Relationship between the results of the absorbance of the WST-8 and MTT assays as a function of the intensity ratio between the bands at about 2920 and 2945 cm^−1^ observed by FTIR for *E. coli* (**a**), *S. epidermidis* (**b**), and *Human fibroblast* (**c**).

**Figure 13:**
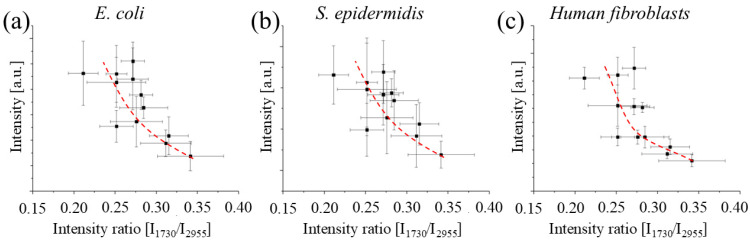
Relationship between the results of the absorbance of the WST-8 and MTT assays as a function of the intensity ratio between the bands at about 1730 and 2955 cm^−1^ observed by Raman spectroscopy for *E. coli* (**a**), *S. epidermidis* (**b**), and *Human fibroblast* (**c**).

Figure 12 and Figure 13 show the relationship between the assay results of Figure 9, Figure 10 and Figure 11 plotted against two different spectroscopic parameters that can be used to estimate the polymerization of the resin, the intensity ratio between the band located at 2920 and 2945 cm^−1^ as observed by FTIR, and the intensity ratio between the band located at 1730 and the band located at 2955 cm^−1^ as measured by Raman spectroscopy. In both cases, we can observe that the survivability of cells and bacteria increases with the polymerization of the resin, which (as seen before) strongly depends on post-curing. But the concerning point is that for both spectroscopic parameters, the toxicity of the resins decrease is slower for cells compared to for bacteria. This indicates that the threshold for achieving a non-cytotoxic resin might be higher than the threshold for achieving an antibacterial one.

## 5. Conclusions

This study explored the interplay between post-curing parameters, mechanical properties, and biological response of a UV-curable resin. We observed that while higher curing temperatures and times improve initial mechanical strength, they can also lead to a decrease later on. Interestingly, incomplete curing exhibited a surprising benefit: an enhanced antibacterial effect against tested bacteria strains (*S. epidermidis* and *E. coli*). However, this came at the cost of increased cytotoxicity towards human cells, mirroring the trend observed with increased curing temperatures.

Our findings also reveal a potential correlation between the intensity ratio of specific Raman bands and the cytotoxicity of the material, highlighting the potential of Raman spectroscopy as a rapid, non-destructive screening tool for assessing the biocompatibility of 3D printed PMMA objects. However, additional studies are required to validate and strengthen this correlation.

Future research should delve deeper into understanding the mechanisms behind the observed relationship between curing parameters, antibacterial effect, and cytotoxicity by studying the interaction of such resins with additional bacterial strains and human cell types. Additionally, exploring alternative resin formulations or post-curing strategies that decouple these effects would be crucial for the development of safe and effective 3D printed materials for biomedical applications.

## Figures and Tables

**Table 1:** Raman vibrational modes observed in Figure 3 and Figure 4.

Position [cm^−1^]	Vibrational Mode	Reference
815	PMMA (C-O-C symmetric stretching)	[28]
990	PMMA (O-C stretching)	[28]
1120	PMMA (C-C stretching)	[28]
1245	UDMA (C-O stretching) PMMA (C-O stretching)	[28,29]
1335	PMMA (CH_2_ twisting or wagging)	[28]
1380	UDMA (C-H)	[29]
1455	UDMA (CH_2_ bending) PMMA (O-CH_3_ bending)	[28,29]
1620	UDMA (C=C)	[29]
1730	UDMA (C=O/C=C conjugation) PMMA (C=O symmetric stretching)	[28,29]
2880	UDMA (CH_2_ asymmetric stretching)	[29]
2930	UDMA (CH_2_ symmetric stretching)	[29]
2955	UDMA (CH_3_ asymmetric stretching) PMMA (α-CH_3_ asymmetric stretching)	[28,29]

**Table 2:** FTIR vibrational modes observed in Figure 5.

Position [cm^−1^]	Vibrational Mode	Reference
1150	UDMA PMMA (C-C-O stretching)	[30,31]
1250	UDMA PMMA (C-O-C stretching)	[30,31]
1290	UDMA (C-O stretching)	[30]
1450	UDMA (CH_2_ scissoring)	[30]
1505	UDMA (CH_2_ bending)	[30]
1715	UDMA (C=O/C=C conjugation) PMMA (C=O)	[30,31]
2880	UDMA (CH_2_ stretching) PMMA (CH_2_ stretching)	[30,31]
2920	UDMA (CH_2_ asymmetric stretching) PMMA (CH_2_ asymmetric stretching)	[30,31]
2945	UDMA (CH_3_ asymmetric stretching) PMMA (CH_3_ asymmetric stretching)	[30,31]

## Data Availability

The raw data supporting the conclusions of this article will be made available by the authors on request.
